# Faster and More Accurate Geometrical-Optics Optical Force Calculation Using Neural Networks

**DOI:** 10.1021/acsphotonics.2c01565

**Published:** 2022-12-19

**Authors:** David Bronte Ciriza, Alessandro Magazzù, Agnese Callegari, Gunther Barbosa, Antonio A. R. Neves, Maria Antonia Iatì, Giovanni Volpe, Onofrio M. Maragò

**Affiliations:** † CNR-IPCF, Istituto per i Processi Chimico-Fisici, I-98158Messina, Italy; ‡ Department of Physics, University of Gothenburg, SE-41296Gothenburg, Sweden; § Universidade Federal do ABC, Av. dos Estados 5001, CEP 09210-580, Santo André, SP, Brazil

**Keywords:** optical tweezers, optical forces, machine learning, Kramer’s rate, ellipsoids

## Abstract

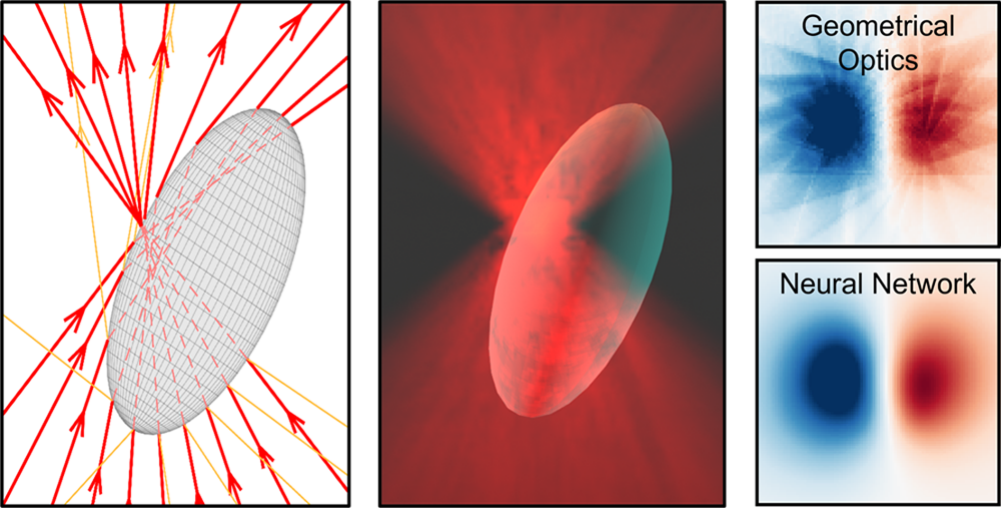

Optical forces are often calculated by discretizing the trapping light beam into a set of rays and using geometrical optics to compute the exchange of momentum. However, the number of rays sets a trade-off between calculation speed and accuracy. Here, we show that using neural networks permits overcoming this limitation, obtaining not only faster but also more accurate simulations. We demonstrate this using an optically trapped spherical particle for which we obtain an analytical solution to use as ground truth. Then, we take advantage of the acceleration provided by neural networks to study the dynamics of ellipsoidal particles in a double trap, which would be computationally impossible otherwise.

## Introduction

Light can exert forces on objects by exchanging momentum with them.^1−3^ Optical tweezers^2,4,5^ use a tightly focused laser beam to trap a particle in three dimensions. Since the pioneering work by Ashkin in the 1970s,^1,4^ they have become a common tool for biology, physics, and nanotechnology.^6−8^ Due to its complexity, the calculation of the forces generated by optical tweezers has often relied on approximations that depend on the size of the particle.^2^ For particles larger than the light wavelength, such as cells,^9,10^ microbubbles,^11^ microplastics,^12^ or metal-coated Janus microparticles,^13^ these forces can be described using the geometrical optics (GO) approximation. In this approximation, the light field is described as a collection of rays and the momentum exchange between the rays and the particle is calculated via the laws of reflection and refraction.^14^

Even though GO force calculations are much faster than solving the full electromagnetic theory, they are still prohibitively slow for many applications. Often, multiple force calculations are required for a single numerical experiment studying the dynamics of a particle in an optical field. For example, to simulate the trajectory of a 2 μm ellipsoidal particle held by a double trap in water that is sufficiently long to estimate its Kramer’s rates, one might require  time steps and therefore force calculations (see Supporting Information, Section 1, “Estimation of the required number of optical force calculations”). Since a single force calculation with sufficient accuracy (i.e., with a large enough number of rays) requires about 0.1 s, it would take several days to obtain one single meaningful trajectory. GO calculations can be sped up by decreasing the number of rays, but this decreases the accuracy.

There are alternatives to increase the speed of the calculation, but they come with their own limitations. The force generated by an optical trap can be approximated by a harmonic potential.^15,16^ However, while this is a good approximation for particles that remain close to the equilibrium point, there are plenty of situations where it is clearly insufficient, e.g., particles escaping an optical trap^17^ or repelled by optical forces.^18^ Another approach could be to avoid the sequential calculation imposed by the random Brownian motion by calculating the force in advance at different points in the parameter space and then interpolating the forces at intermediate points.^19^ This improves the calculations for a sphere moving in 3 dimensions where a grid of 100^3^ points would suffice. However, the number of points that need to be stored in memory grows exponentially with the number of degrees of freedom (DOF), and as we consider more complex shapes and configurations, the required grid points would easily surpass the current computer memory storage capabilities (e.g., the position, orientation, size, and aspect ratio of an ellipsoid of revolution requires 7 DOF).

Recently, neural networks (NNs) have been demonstrated to be a promising approach to improve the speed of optical force calculation for spheres using the T-matrix method.^20^ NNs are able to use data to adapt their solutions to specific problems.^21^ These algorithms have proved to improve on the performance of conventional ones in tasks such as determining the scattering of nanoscopic particles,^22^ enhancing microscopy,^23^ tracking particles from digital video microscopy^24^ or even epidemics containment.^25^

In this study, we show that NNs can be used to accelerate the force calculations, while also surprisingly improving the accuracy of GO. We have first demonstrated this for a spherical particle with 3 DOF, corresponding to the position of the particle, when compared to our analytical solution for the optical force applied on a sphere by a focused beam. Then, we expand the work to 9 DOF by including all the relevant parameters for an optical tweezers experiment such as refractive index, particle shape, particle position, and numerical aperture of the objective. Finally, we study the dynamics of ellipsoidal particles in a double beam configuration by exploiting our NNs as a tool to map fast and accurately the parameter space, a relevant task that would be computationally impossible otherwise.

## Results

We employ NNs to calculate optical forces in three different study cases. First, we compare the traditional GO calculation to the NNs approach in the simplest case of a sphere in an optical trap (3 DOF), where we have developed an analytical solution that we can employ as ground truth, based on Ashkin’s original contribution but considering a continuous distribution of rays instead of a discrete set. Second, we expand this to the case of an ellipsoid (9 DOF), increasing the number of DOF to a value sufficient for most situations people encounter when working with optical tweezers. In these two study cases, we show how NNs are not only much faster but also more accurate than GO. Finally, we use this last NN to explore the dynamics of ellipsoids in a double beam optical tweezers, a problem that would have been computationally impossible to tackle with the conventional approach.

### Sphere in a Single Trap

**Figure 1:**
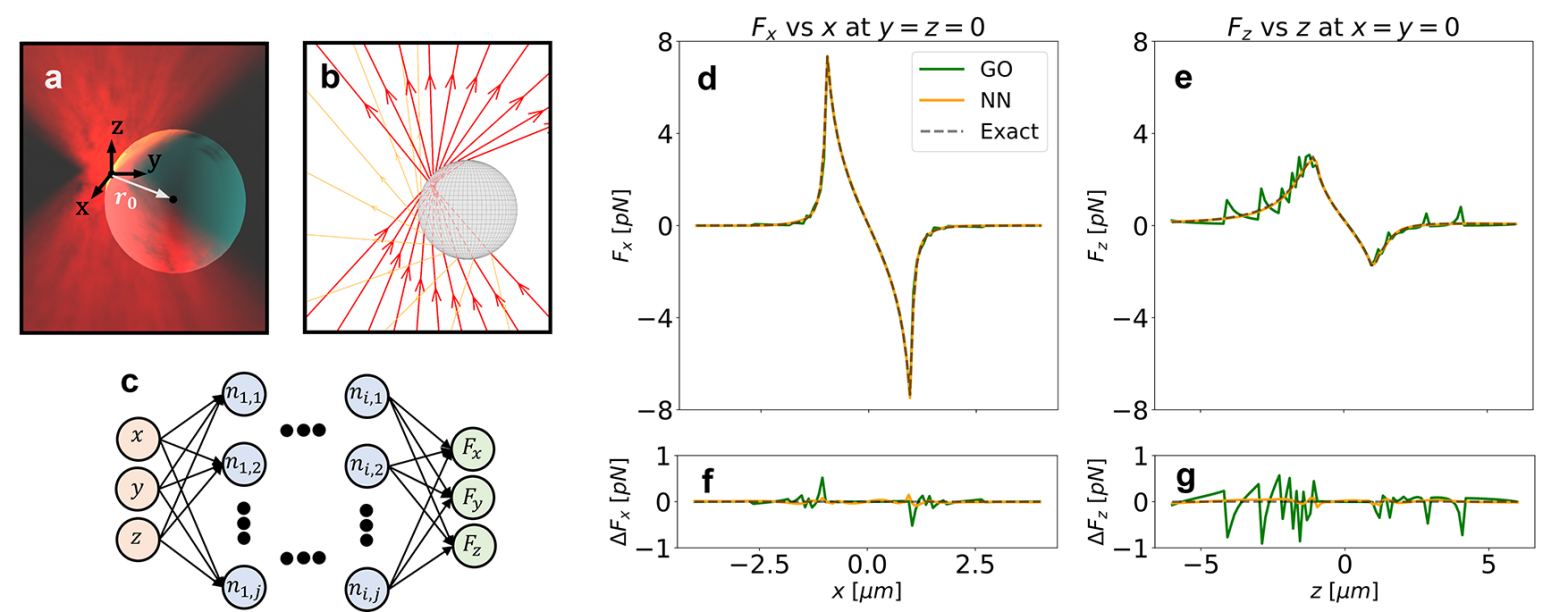
Optical force calculations on a sphere. (a) 3D schematic of the sphere in an optical trap. (b) GO schematic of the rays reflected and transmitted by the sphere. (c) Architecture of a densely connected NN with an input layer (light red, particle position: *x*, *y*, *z*), an output layer (light green, optical force: *F*_*x*_, *F*_*y*_, *F*_*z*_), and *i* hidden layers (light blue) in between. Each of the hidden layers has *j* neurons, and all the neurons in each layer are connected to all the neurons in the previous and next layer. In the model trained with 100 rays, *i* = 5 and *j* = 16. (d,e) Optical force along the (d) *x*-axis and (e) *z*-axis calculated using GO (green solid line) and NN (orange solid line), as well as exact model (black dashed line) obtained using eq 2. (f,g) The difference between the exact model and the GO (green lines) and NN (orange lines) calculations along the two axes shows that the NN is more accurate than GO, especially for *F*_*z*_ where the GO artifacts are more evident.

We start by studying the simplest case: we calculate the forces (*F*_*x*_, *F*_*y*_, *F*_*z*_) applied by an optical tweezers on a sphere as a function of its position (*x*, *y*, *z*), see Figure 1(a). We repeat this calculation with two different methods and compare them with the exact analytical calculation. First we employ the conventional GO approach considering 100 rays (Figure 1(b)). Second, we use these data generated with GO to train a NN with 3 inputs, 3 outputs, and 5 hidden layers in between ( trainable parameters, see Figure 1(c) and Supporting Information, Section 3, “Neural Networks” for more details about its architecture). The parameters of the system are typical of an optical tweezers experiment: 2 μm sphere with refractive index 1.5 in water, objective’s numerical aperture (NA) 1.3, and laser power 5 mW.

The NN provides more accurate results than GO for the same number of rays. Both GO and NN calculations show the expected equilibrium position close to the focus for both transversal (*x*) and axial (*z*) directions, see Figure 1(d,e). However, GO introduces artifacts due to the discretization of the continuous light beam into a finite number of rays, see Figure 1(a,b). We manage to remove the artifacts by designing a NN that is complex enough to learn the smooth force profile, but not the superimposed fluctuating artifacts. This strategy allows the NN to achieve an accuracy higher than that of the training data, see Figure 1(f,g).

**Figure 2:**
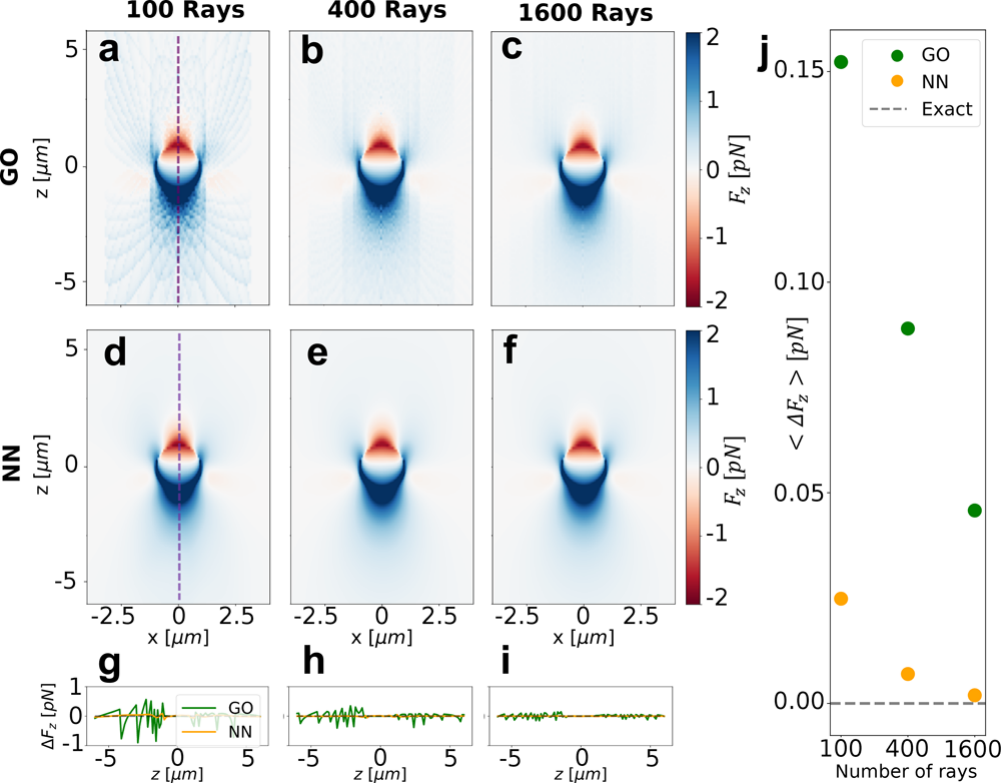
Comparison of GO and NN for different numbers of rays. (a,b,c) GO calculation of *F*_*z*_ in the *xz*-plane. The number of rays considered for each calculation is 100, 400, and 1600 respectively. (d,e,f) NN predictions when trained with data generated with 100, 400, and 1600 rays, respectively. (g,h,i) Difference between GO and NN, and the exact model across the axis *y* = *x* = 0 (dashed region in (a) and (d)). (j) Average error of GO and NN with the exact model in the calculation of *F*_*z*_ across the axis *x* = *y* = 0 for 100, 400, and 1600 rays. The NN is always more accurate than GO for an equivalent number of rays. Furthermore, even the NNs trained with the least amount of rays (100) are more accurate than GO with the most amount or rays (1600).

We can improve the accuracy of GO by increasing the number of rays. To illustrate this, we now focus on the axial force *F*_*z*_ (light going toward positive *z*) across the *xz*-plane. Figure 2(a–c) shows the force calculation with GO for different number of rays. All the calculations retrieve the expected result of an equilibrium point close to the focus, positive force (blue) below the focus, and negative force (red) over the focus. However, there are some artifacts that depend on the number of rays and that affect the accuracy of the calculation. Comparing the GO calculations with the analytical ground truth (eq 2), we obtain the anticipated results: a higher number of rays result in a lower error (see Figure 2(g–i) where the solid green line corresponds to the error of GO against the exact analytical model). On the other hand, the NN (Figure 2(d–f)) provides more accurate results than GO even when trained with data obtained with a lower number of rays (Figure 2(g–i) where the solid orange line represents the error of the NN). Furthermore, compared with our exact solution across the *z*-axis, even the NN trained with 100 rays is more accurate than the GO considering 1600 rays, see Figure 2(j).

The NN is not only more accurate (Figure 2), but also much faster than GO. GO reaches a calculation speed of around 50 calculations per second when considering 100 rays, and this speed decreases down to 17 calculations per second for 1600 rays. The calculation speed by using our trained NN is between 1 and 2 orders of magnitude faster, see Table 1. The calculation speed of the NN does not depend on the number of rays used in the training set, but on its architecture and on its number of trainable parameters (see Supporting Information, Section 3, “Neural Networks”). If we consider many particles, many beams, or we run many simulations at the same time, we can benefit from the straightforward implementation of the NN in the GPU to increase the speed by another 2 orders of magnitude.

**Table 1:** Calculations Per Second for the Sphere with 3 DOF

	GO	NN (CPU)	NN (GPU)
100 rays	50.4 ± 0.5	407 ± 2	54 100 ± 300
400 rays	32.1 ± 0.3	405 ± 2	54 400 ± 200
1600 rays	16.8 ± 0.1	532 ± 3	59 700 ± 400

### Ellipsoid in a Single Trap

**Figure 3:**
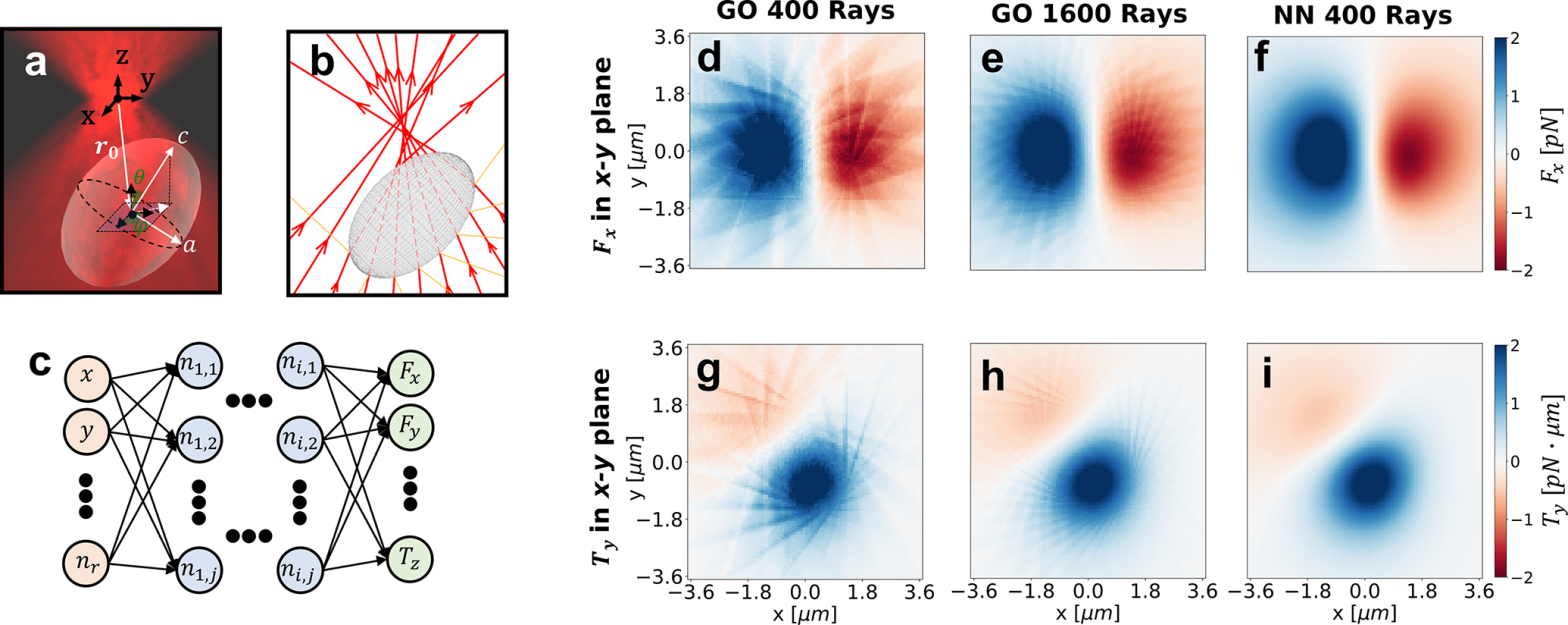
Optical forces calculations for an ellipsoid. (a) 3D schematic of the ellipsoid in an optical trap. (b) GO schematic of the rays reflected and transmitted by an ellipsoid. (c) Architecture of a densely connected NN with an input layer (light red), an output layer (light green), and *i* hidden layers (light blue) in between. Each of the hidden layers has *j* neurons, and all the neurons in each layer are connected to all the neurons in the previous and next layer. In both NNs *j* = 384, but the for the one trained with 400 rays *i* = 5, while in the one with 1600 rays *i* = 8. (d–i) GO and NN calculations of *F*_*x*_ (d,e,f) and *T*_*y*_ (g,h,i) in the *xy*-plane at *z* = −3.0 μm. The parameters have been selected randomly across the space of parameters for which we have trained the NN. The major semiaxis (*c*) of the ellipsoid is 3.7 μm long, the aspect ratio (AR) is 1.5, and its orientation is determined by θ = 1.03 rad and ϕ = 2.14 rad. The refractive index (*n*_p_) of the particle is 2.5, and the numerical aperture of the objective 1.2.

We now consider a more complex case with more DOF: We include different positions (*x*, *y*, *z*), orientations (θ and ϕ, corresponding to the angle of the major axis with the *z* direction and to the angle between the *x* direction and the projection of the major axis in the *xy*-plane), length of the major axis (*c*), aspect ratios (AR), refractive indices (*n*_p_) of the particle, and different numerical apertures of the objective (NA), see Figure 3(a). The forces and torques are computed using GO considering 400 and 1600 rays, see Figure 3(b). The data generated with GO are used to train a NN with 9 inputs (corresponding to the 9 DOF) and 6 outputs (*F*_*x*_, *F*_*y*_, *F*_*z*_, *T*_*x*_, *T*_*y*_, *T*_*z*_), see Figure 3(c). The architecture and the range of validity of the NN are defined in Supporting Information, Section 3, “Neural Networks”. To account for the higher complexity of the problem, the training data are increased up to 2.5 × 10^7^ points, larger than for the sphere but much smaller than the prohibitive  points that would have been required for the interpolation approach. The NN trained with data generated with 1600 rays has more trainable parameters, so it can benefit from the increased accuracy.

Similarly to what we observed for the sphere, the NN improves the accuracy and drastically increases the speed when compared to GO. Even though in this situation we do have no ground truth to compare the accuracy of the different methods as there is no equivalent for ellipsoids of eq 2, we can compare the results with 400 rays against those with 1600 rays. Differently from the case of the sphere, we can now explore all the 9 DOF. Selecting a random *xy*-plane in the 9 DOF space of parameters, the NN obtains the expected profile of the forces, see Figure 3(d–f), and the torques, see Figure 3(g–i). Note that there is a nonzero torque at *x* = *y* = 0 because the major axis of the ellipsoid is not aligned with the beam. The NN overcomes the accuracy of the training data even when trained with only 400 rays. Like in the previous example, the NN improves the calculation speed by 1–2 orders of magnitude when using the CPU and two more orders of magnitude when using the GPU (see Table 2).

**Table 2:** Calculations Per Second for the Ellipsoid with 9 DOF

	GO	NN (CPU)	NN (GPU)
400 rays	9.62 ± 0.06	404 ± 1	50 200 ± 300
1600 rays	5.59 ± 0.02	297 ± 1	43 400 ± 1400

### Ellipsoid in a Double Trap

We can now explore the dynamics of an ellipsoid in a double trap by enhancing the calculation using the previously described NN. In a microscopic system, transitions between different equilibrium points can be induced by thermal fluctuations that allow the system to overcome the potential barrier. These transitions play a key role in electronics,^26^ physics,^27^ and biology,^28^ and optical tweezers have become a useful tool to study them.^29−32^ These previous studies have focused on spherical particles, considering different shapes could enrich the dynamics of these systems. However, these simulations often require a lot of repetitions of the force calculation, which with the conventional GO becomes prohibitively slow. In this situation, traditional approaches to speed up the calculation become unfeasible. We cannot consider the interpolation approach due to the high number of DOF of the system, and we cannot use the harmonic approximation because of the broken assumption of small displacements around the equilibrium point. Therefore, we employ our trained NN to overcome these issues and achieve a fast and accurate calculation of optical forces. Since there are two focused beams, we first calculate the force and torque applied by each of them using the trained NN and then add both contributions to obtain the total effect on the particle. See Supporting Information, Section 4, “Simulation of the Brownian dynamics” for details about the simulation of the dynamics.

**Figure 4:**
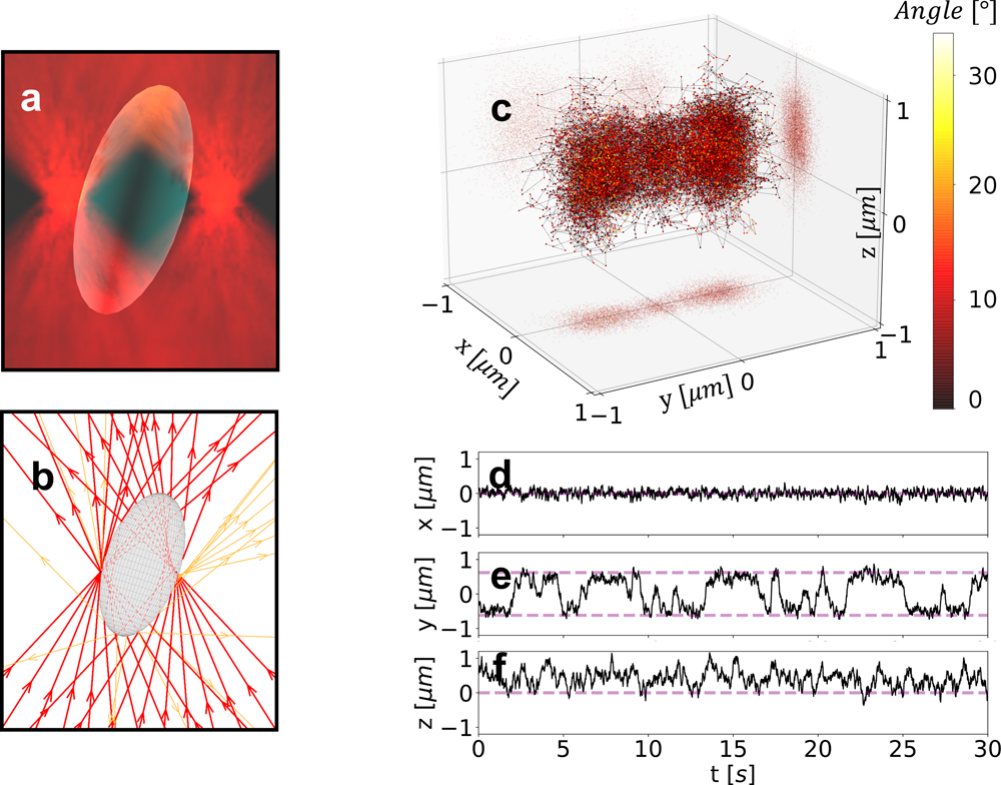
Simulation of the dynamics of an ellipsoid in a double trap. (a) 3D schematic of an ellipsoid in a double trap. (b) GO schematic of the rays reflected and transmitted by an ellipsoid in a double trap. (c) Simulated 2 min trajectory of an ellipsoid in a double trap. The color codes the orientation of the long axis of the ellipsoid with respect to the beam. The ellipsoid has a refractive index of 1.5, its major semiaxis is 4.2 μm, and its short semiaxis is 1.5 μm. The distance between the two beams is 1.24 μm, the intensity of each of them is 0.25 mW, and the NA of the objective focusing the light is 1.30. (d,e,f) A 20-s trajectory of the center of mass along the *x*-, *y*-, and *z*-direction, respectively. The dashed purple lines correspond to the position of the focus of the beams in each of the axes.

On the single-trajectory level, we observe the expected results for the dynamics of an ellipsoid in a double trap (Figures 4(a,b)). The particle remains with its long axis aligned along the direction of the beam (color coding of Figure 4(c)), which is typical for this kind of elongated structure.^33,34^ Apart from the focuses of the two traps, an additional equilibrium point emerges in between (densely explored region around *x* = *y* = 0 in Figure 4(c)). Furthermore, when looking at the trajectories (Figure 4(c–f)), the particle center remains confined around the origin of the *x*-axis (as expected), jumps between the two traps and an intermediate equilibrium point along the *y*-axis, and it is slightly displaced toward the positive values of *z*-axis due to the scattering force as it has already been observed in the literature.^29^

**Figure 5:**
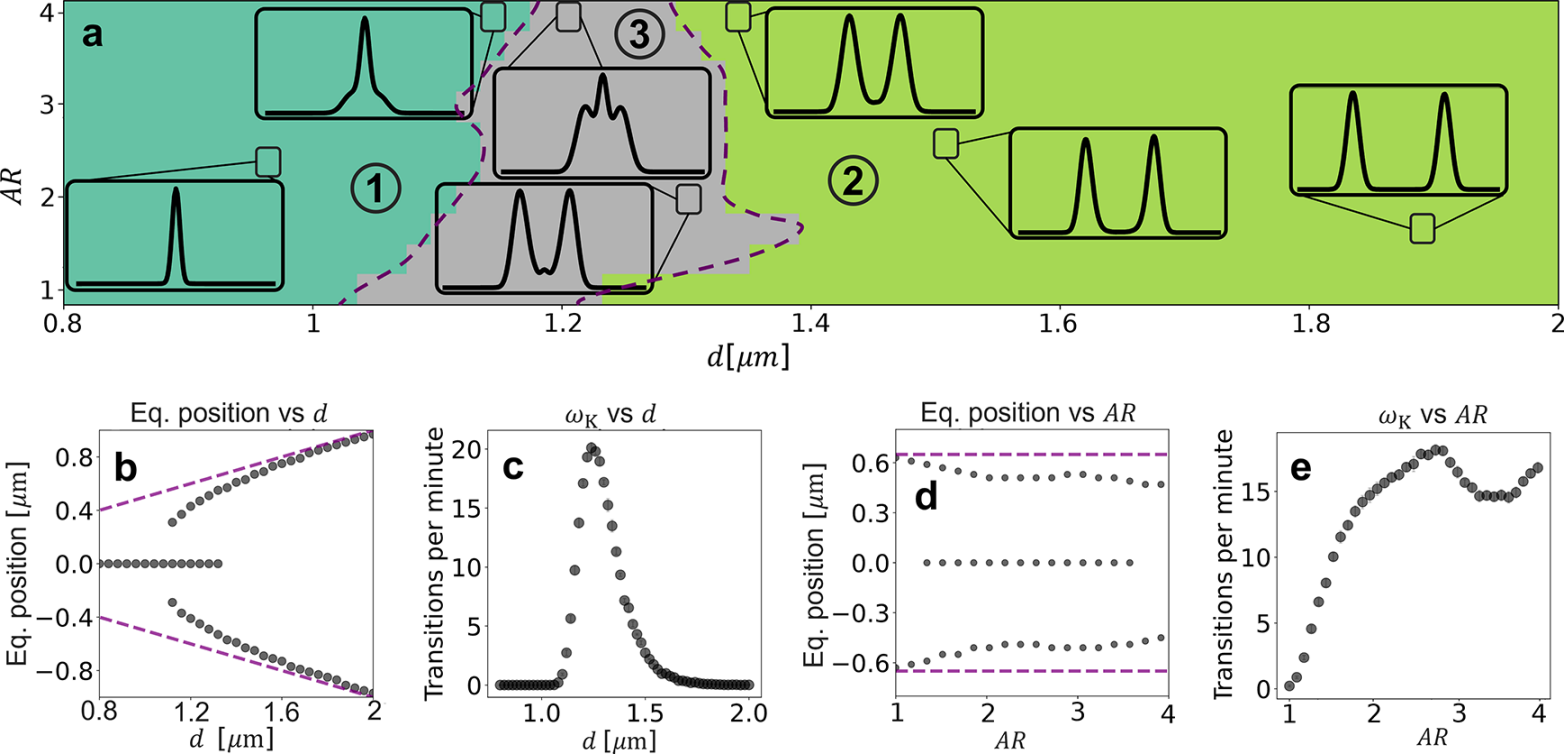
Dynamics of an ellipsoid in a double trap changing the aspect ratio (AR = *c*/*a*) and the distance between traps (*d*). NA = 1.3, *n*_p_ = 1.5, *a* = *b* = 0.75 μm are kept constant over the simulation, the particle is in water at 20 °C, and the intensity of each beam is 0.25 mW. Notice that while the length of the axes *a* and *b* is fixed, as we change *c* and the AR we also change the volume of the ellipsoid. (a) State diagram in the AR–*d* parameter space. The parameter *d* samples the space from the situation where the two traps behave as one to the situation where the two traps are completely independent of each other. The AR ranges from 1 (a sphere) to 4 (ellipsoid). The three colored regions correspond to 1 (blue), 3 (gray), and 2 (green) equilibrium points, and the purple dashed line indicates the transitions between regions. The insets show the probability distribution averaged over 100 trajectories. In (b,c) we study the situation where AR is fixed to 2.8 and we change *d*, while in (d,e) *d* is kept constant to 1.3 μm and we vary the AR. (b) Position of the equilibrium points vs *d*. The purple dashed line indicates the trap position. (c) Kramer’s rate (ω_K_) vs *d*. (d) Position of the equilibrium points vs AR. The purple dashed line indicates the trap position. (e) ω_K_ vs AR.

Powered by the fast NN calculation, we can simulate many trajectories as the ones presented in Figure 4 and explore the statistical properties of the dynamics. Exploring different configurations of parameters, we study how the equilibrium points and the Kramer’s rate (ω_K_) depend on the aspect ratio (AR) and on the distance between traps (*d*). We focus first on the dependence with *d*. Regarding the equilibrium points, in the state diagram we can distinguish three different regions, see Figure 5(a). When the traps are close to each other they behave as a single one with the particle trapped in between. By increasing the separation between traps (*d*), the probability distribution starts widening until reaching a region with 3 equilibrium points. Separating even further the traps, the intermediate equilibrium position disappears and eventually the traps behave independently. The behavior of the ellipsoids (Figure 5(b)) is very similar to what was predicted and observed for spheres.^30^ Regarding the dependence of ω_K_ with *d*, the transition rate reaches a maximum in the region where the system transits from three to two equilibrium points, see Figure 5(c). We now focus on the dependence of the equilibrium points and ω_K_ on AR, i.e., understanding how the change in shape affects the dynamics of the particle. Fixing *d* = 1.3 μm, the two farthest equilibrium points come closer to each other when increasing the length of the ellipsoid. Moreover, a third equilibrium point emerges for an intermediate region of lengths, see Figure 5(d). Studying ω_K_, it increases with the length of the ellipsoid until reaching a maximum and remaining approximately constant, see Figure 5(e). It is known that the stiffness of the trap in the beam direction decreases with the length for elongated structures.^35−37^ This decrease in the stiffness (see Supporting Information, Section 6, “Trap stiffness dependence on the aspect ratio”) makes the particle more likely to reach the transition region as described for spheres in ref (29), and therefore the Kramer’s rate increases.

It is worth noticing that while having a NN with many DOF is useful to approach systems and study their dependence on different parameters, the increase in generality comes with a slight decrease in accuracy and calculation speed (still outperforming GO) and a longer training (requiring more data). This means that we have not employed the full potential of the NN in the study of the dynamics of the ellipsoid in a double trap, as we have kept some input parameters of the NN constant (NA, *a*, and *n*_*p*_). However, even in the nonoptimal situation, we have proved that it is possible to train a single NN able to account for all the DOF of a typical OT experiment and that it allows the study of new problems. If the reader wants to consider other specific situations where most of the DOF remain fixed, it might be beneficial to design more specific NNs. The trained NNs and a tutorial to show how to use them have been prepared and made available (see the Data Availability Statement).

## Conclusions

Employing NNs, the compromise between speed and accuracy for the calculation of optical forces on microscopic sized particles is no longer a limitation. By computing the optical forces using GO, it is possible to train a NN that predicts the forces not only faster but also with higher accuracy. The fact that it can increase the accuracy of the training data allows us to perform the training with low accuracy data that are generated faster, requiring only a small set of more accurate data to trigger when to stop the training.

The NN approach is not limited to spheres, but a single NN trained to include as DOF all the relevant parameters in a basic optical tweezers experiment still outperforms the speed and accuracy of GO. This enhancement allows the computation of the dynamics of ellipsoids in a double beam optical tweezers where we studied the equilibrium points and the Kramer’s rate as a function of the distance between traps and the aspect ratio of the ellipsoids. Even though with the conventional GO approach this could have been done for a single point in the AR–*d* parameter space, mapping the full space was unfeasible.

While the process of obtaining a trained NN can be time-consuming, the advantages of a trained NN are many. The most time-consuming step is generating the training data. However, this computation does not need to be sequential (as it is the case for the Brownian dynamics simulation), and it can be sped up by parallelizing the calculation. Once the NN has been trained there are two main advantages. On the one hand, the increase in speed allows the exploration of situations that remained out of the scope of the GO approach. On the other hand, a trained NN is easier to use and to couple to other programs than the existing GO softwares. We have prepared and made available a tutorial where we include the trained NNs and illustrate how you can use them (see the Data Availability Statement). Note that even though we have considered a basic OT experiment, this approach can be expanded to different trapping configurations, beam profiles, or shape of particles without an increase in complexity (if not increasing the DOF). We believe that NNs could democratize the ability to perform optical forces calculations, allowing for a further development of the optical manipulation field pushed by numerical simulations.

## Methods

### Geometrical Optics

Geometrical optics (GO) is an approach that describes the propagation of light in terms of rays. A ray incident (direction ) on a particle undergoes an infinite number of scattering events (as shown in Supporting Information, Figure S1). In each scattering event, the ray hits the surface separating the particle (refractive index *n*_p_) from the surrounding medium (refractive index *n*_i_), and is partly reflected and partly transmitted. In our study we have considered that the incoming rays are circularly polarized, which in GO is equivalent to nonpolarized light. The force acting on the particle is equal and opposite to the change in momentum of the light, i.e., the momentum of the incident light minus the momenta of the reflected ray in the first scattering event (direction ) and the transmitted rays in all subsequent scattering events (direction  where *s* > 1 is the number of the scattering event). Therefore, the force of a single ray on a particle^14,38,39^ is
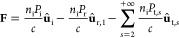
1

When calculated numerically, this sum is truncated. We set the truncation error on the power at 10^–12^, i.e., we stop when the residual power falls below 10^–12^ of the original power, and consequently the relative error on the calculated quantity (i.e., the force in this case) is less that 10^–12^. This inevitably introduces some numerical errors even though the ray power quickly decreases. To calculate the force generated by a focused laser beam, the beam is split into a set of rays—the higher the number of rays, the more accurate the calculation becomes, but also the longer it takes. To compute this force we use the computational toolbox OTGO.^14^

### Exact Calculation

In order to have a ground truth independent of the numerical GO calculations, we have derived an analytical solution for the optical force applied on a sphere by a focused beam (see Supporting Information, Section 2, “Exact force calculation in GO”), building on the analytical formula obtained by Ashkin for the force applied by a circularly polarized ray on a sphere.^38^ A ray with power d*P* incident onto a sphere at an incident angle σ is partly reflected and partly transmitted according to the known Fresnel coefficients. By determining the weight of each ray within the beam intensity profile and integrating over a continuous distribution of rays, we have the analytical solution for the transversal and axial axes.
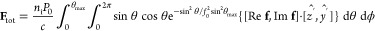
2where
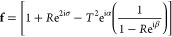
3is the complex force term for a single ray. *R* and *T* are the reflection and transmission coefficients for circularly polarized light (see Supporting Information, Section 2, “Exact force calculation in GO”), σ and *r* are the angles of incidence and refraction, related by Snell’s law: *n*_i_ sin σ = *n*_p_ sin *r* (see Supporting Information. Figure S1) and α = 2σ – 2*r*, β = π – 2*r*. This expression establishes a ground truth that is free of the artifacts introduced by the discretization into a finite number of rays (see Supporting Information, Section 2, “Exact force calculation in GO”) and can therefore be employed to check the accuracy of the solutions obtained using GO and NNs.

### Neural Networks

We use NNs to predict the optical forces in the GO approximation. NNs are supervised machine-learning algorithms that learn from a set of data to model relationships between the input features (e.g., relevant parameters of a particle in an optical tweezers) and the target prediction output (e.g., force applied by the optical tweezers). We have employed fully connected NNs because they have already proved successful in similar situations.^20^ The NNs have been trained using data generated with GO using the toolbox OTGO.^14^ Even though the training data come with artifacts due to the finite number of rays, both the NN architecture and the training process are designed to obtain NN predictions that get rid of these artifacts. Therefore, we designed complex enough NN architectures to learn the force profile, but not so complex to learn the artifacts. Furthermore, we employ a control data set generated with a higher number of rays and stop the training once the error against this control starts increasing. For a detailed explanation of the training, we refer the reader to the Supporting Information, Section 3, “Neural Networks”.

## Data Availability

The NNs used to obtain these results and a tutorial about how to use them and train similar ones are available at: https://github.com/brontecir/Deep-Learning-for-Geometrical-Optics.

## Supporting Information Available

Estimation of the required number of optical forces calculations, detailed explanation of the exact force calculation in the GO approximation, description of the NNs architecture and training, explanation of the method to compute the accuracy and speed of the different calculation approaches, and calculation of the trap stiffness dependence on the aspect ratio of an ellipsoid (PDF)
