# Supplementary material for: Faster and More Accurate Geometrical-Optics Optical Force Calculation Using Neural Networks
Source: ACS Photonics. 2022 Dec 19;10(1):234–41. doi: 10.1021/acsphotonics.2c01565 (PMC9853855; doi:10.1021/acsphotonics.2c01565)
Supplement: Supplementary file 1 — ph2c01565_si_001.pdf [file ph2c01565_si_001.pdf]

Supporting Information. Faster and more accurate geometrical-optics optical force calculation using neural networks

David Bronte Ciriza,*,† Alessandro Magazzù,† Agnese Callegari,‡ Gunther Barbosa,¶ Antonio A. R. Neves,¶ Maria Antonia Iatì,† Giovanni Volpe,*,‡ and Onofrio M. Maragò†

†*CNR-IPCF, Istituto per i Processi Chimico-Fisici, I-98158 Messina, Italy*

‡*Department of Physics, University of Gothenburg, SE-41296 Gothenburg, Sweden*

¶*Universidade Federal do ABC, Av. dos Estados 5001, CEP 09210-580, Santo André, SP, Brazil*

E-mail: brontecir@ipcf.cnr.it; giovanni.volpe@physics.gu.se

Number of pages: 17

Number of tables: 1

Number of figures: 3

1. Estimation of the required number of optical force calculations

Consider a $2\,\mu$m polystyrene ellipsoidal particle in a double beam in water. We can assume that the system is in the low Reynolds number regime and linearizing the trap force around their respective equilibrium point, the optical contribution of each trap to the motion can be expressed as $\Delta x = \frac{kx}{\gamma}\Delta t$ where the characteristic time of the optical trap can be defined as $\tau_{OT} = \gamma/k$. Realistic values of the spring constant k are around $50\,\text{pN}/\mu\text{m}$[1] and at lab temperature, the water viscous coefficient γ is about $20\,\text{pN}\cdot\text{ms}/\mu\text{m}$, giving a characteristic time of around $400\,\mu$s. For an accurate simulation, it is reasonable to aim for a time step one order of magnitude smaller than the characteristic time, imposing a time step of $40\,\mu$s, therefore a sampling rate of 25000 fps (if we were conducting an experiment). If the Kramer's transition[2] takes tens of seconds, one would need to compute a hundreds of seconds trajectory, considering two beams, the required number of force calculations is around $1\cdot 10^7$.

2. Exact force calculation in the geometrical optics approximation

Considering a beam formed by a superposition of circularly polarized rays, each ray with power ($\mathrm{d}P$) incident into a sphere at an incident angle (σ). Each ray is partially reflected (R), and transmitted (T) according to the Fresnel coefficients red for circularly polarized light described as:

$$R(\sigma) = \frac{1}{2}\left\{\left(\frac{\sin(\sigma - r)}{\sin(\sigma + r)}\right)^2 + \left(\frac{\tan(\sigma - r)}{\tan(\sigma + r)}\right)^2\right\} \tag{1}$$

$$T(\sigma) = 1 - R(\sigma) \tag{2}$$

where σ and r are the angles of incidence and refraction, related by Snell's law.

The optical forces on the sphere along the beam axis (z) and transverse direction (y) can be derived from,[3] where the values of the force in both axes are the real and imaginary part respectively of the following expression:

$$\mathbf{F}_{\text{ray}} = \frac{n_i dP}{c} \left\{ 1 + Re^{2\text{i}\sigma} - T^2 \text{e}^{\text{i}\alpha} \left[\frac{1}{1 - Re^{\text{i}\beta}} \right] \right\} \tag{3}$$

where n_i is the external medium, $\alpha = 2\sigma - 2r$, $\beta = \pi - 2r$, where σ and r are the angles of incidence and refraction, related by Snell's law $n_i \sin\sigma = n_p \sin r$. By integrating over the continuous distribution of rays that reach the particle, an analytical expression for the force could be retrieved.

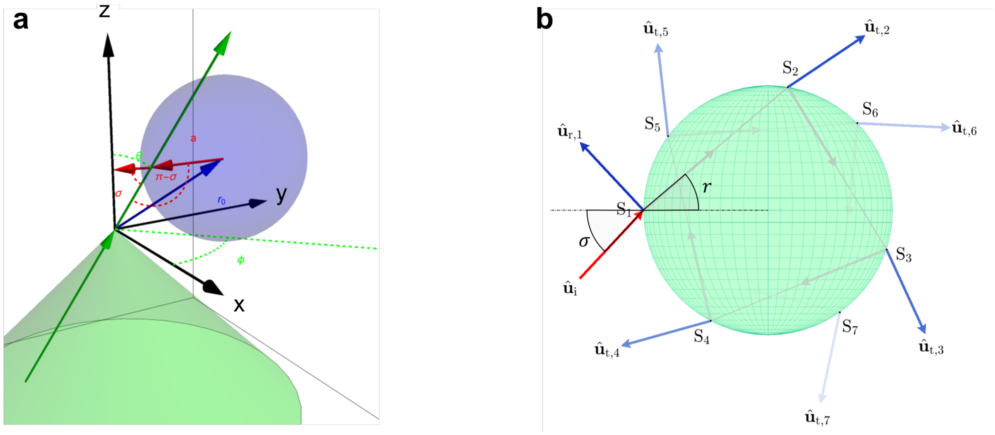

Fig. S1: Schematics of the geometry and the ray scattering events. (a) Geometry and parameters considered in the analytical calculation. (b) Scattering events of a ray on a sphere. The reflected, transmitted and refracted rays are shown.

Focusing bundle of rays

In an optical tweezers, there is not a single ray but a bundle of focused rays. We therefore map the directions of the incident rays to the incidence angle onto the sphere.[4–6] For the incident beam we consider converging rays with angles from $\theta = 0$ to $\theta_{\max}$ that is defined by the numerical aperture of the system.

An aplanatic focusing system needs to satisfy the sine condition and the intensity law. The sine condition indicates that each incoming ray at a height ρ from the optical axis,

when intercepting a sphere of radius f converges to the focus, resulting in $\rho = f \sin\theta$. The intensity law is due to energy conservation, such that the beam power before and after the focusing objective must be the same. The resulting weighted power for each converging ray in spherical coordinates is

$$\mathrm{d}P = I_0 \mathrm{e}^{-2f^2 \sin^2\theta/w^2} f^2 \sin\theta \cos\theta \,\mathrm{d}\theta \,\mathrm{d}\phi \tag{4}$$

where w denotes the beam-waist radius of the laser beam before the objective. Considering each ray carrying $\mathrm{d}P$ of power and following the expression and the convention for the real and imaginary part explained in Eq. 3, the total force applied by all the rays in the beam could be obtained from,

$$\mathbf{F}_{\mathrm{tot}} = \frac{2\pi n_i I_0 f^2}{c} \int_0^{\theta_{max}} \left\{ 1 + R\mathrm{e}^{2\mathrm{i}\sigma} - T^2 \mathrm{e}^{\mathrm{i}\alpha} \left[\frac{1}{1 - R\mathrm{e}^{\mathrm{i}\beta}} \right] \right\} \mathrm{e}^{-2f^2 \sin^2\theta/w^2} \sin\theta \cos\theta \,\mathrm{d}\theta \tag{5}$$

Displacing the sphere from beam focus

Let us consider the coordinate system whose origin is centered at the beam focus.[7] The beam has incoming rays towards the positive z-axis, each of which is characterized by the spherical coordinates (θ,ϕ). The unit incident ray by $\hat{\mathbf{i}}$ is,

$$\hat{\mathbf{i}} = \sin\theta \cos\phi \,\hat{\mathbf{x}} + \sin\theta \sin\phi \,\hat{\mathbf{y}} + \cos\theta \,\hat{\mathbf{z}} \tag{6}$$

The sphere center is arbitrarily located at $\mathbf{r_0}$ with respect to the coordinate system as,

$$\mathbf{r_0} = x_0 \,\hat{\mathbf{x}} + y_0 \,\hat{\mathbf{y}} + z_0 \,\hat{\mathbf{z}} \tag{7}$$

or in polar coordinates as,

$$\mathbf{r_0} = \rho_0 \cos\phi_0 \,\hat{\mathbf{x}} + \rho_0 \sin\phi_0 \,\hat{\mathbf{y}} + z_0 \,\hat{\mathbf{z}} \tag{8}$$

where ρ_0 is the radial distance with the z axis, ϕ_0 is the angle with the x direction and z_0 is the position in the z axis.

The equation for the sphere would be $(x-x_0)^2+(y-y_0)^2+(z-z_0)^2 = a^2$, where a is the radius. To determine the point of intercept for an incident ray onto an arbitrarily positioned sphere, we can parameterize the incident ray as

$$\mathbf{d} = t\hat{\mathbf{i}} = t\left(\sin\theta\cos\phi\,\hat{\mathbf{x}} + \sin\theta\sin\phi\,\hat{\mathbf{y}} + \cos\theta\,\hat{\mathbf{z}}\right) \tag{9}$$

where t is a scalar that would be positive if it intercepts the sphere above the origin and negative if it intercepts it below.

A third vector $\mathbf{a}$, which leads from the center of the sphere to the surface point where the incident ray intercepts has modulus equal to it radius a. This vector can be determined relating it to the other vectors, as

$$\mathbf{d} = \mathbf{r_0} + \mathbf{a} \tag{10}$$

All three vectors in Eq. 10 are in the plane of incidence. We shall derive two cosine law relations from equations,

$$\mathbf{r_0} = \mathbf{d} - \mathbf{a} \tag{11}$$

$$\mathbf{a} = \mathbf{d} - \mathbf{r_0} \tag{12}$$

By squaring Eqs. 11 and 12 we have,

$$\begin{aligned} r_0^2 &= t^2 + a^2 - 2|t|a\cos(\pi-\sigma) \\ r_0^2 &= t^2 + a^2 + 2ta\cos(\sigma) \end{aligned} \tag{13}$$

and

$$a^2 = [t\,(\sin\theta\cos\phi\,\hat{\mathbf{x}} + \sin\theta\sin\phi\,\hat{\mathbf{y}} + \cos\theta\,\hat{\mathbf{z}}) - (\rho_0\cos\phi_0\,\hat{\mathbf{x}} + \rho_0\sin\phi_0\,\hat{\mathbf{y}} + z_0\,\hat{\mathbf{z}})]^2$$
$$a^2 = (t\sin\theta\cos\phi - \rho_0\cos\phi_0)^2 + (t\sin\theta\sin\phi - \rho_0\sin\phi_0)^2 + (t\cos\theta - z_0)^2 \quad (14)$$
$$a^2 = t^2 + r_0^2 - 2t\rho_0\sin\theta\cos(\phi-\phi_0) - 2tz_0\cos\theta$$

By isolating $r_0^2 - a^2$ from the above two equations (Eq. 13 and 14), we have

$$\cos(\sigma) = \frac{\rho_0}{a}\sin\theta\cos(\phi-\phi_0) + \frac{z_0}{a}\cos\theta - \frac{t}{a} \quad (15)$$

To further simplify the above relation we need the value for the t-parameter. This is obtained from Eq. 14.

$$t = \rho_0\sin\theta\cos(\phi-\phi_0) + z_0\cos\theta \pm \sqrt{(\rho_0\sin\theta\cos(\phi-\phi_0) + z_0\cos\theta)^2 - (r_0^2 - a^2)} \quad (16)$$

Not all incident rays will contribute to the optical force. To consider only the rays that are incident upon the sphere and refracts, this has to intercept the spherical particle in two points. For this to occur, the term inside the square-root of the above equation needs to be positive. With two solutions for the t–parameter we choose the one with the negative sign, corresponding to the ray that first intercepts the sphere. Substituting this t-value in the equation for the cosine of the incident angle, we get

$$\cos(\sigma) = +\sqrt{\left(\frac{\rho_0}{a}\sin\theta\cos(\phi-\phi_0) + \frac{z_0}{a}\cos\theta\right)^2 - \left(\left(\frac{r_0}{a}\right)^2 - 1\right)} \quad (17)$$

Mapping the resulting 2D forces in the plane of incidence to our coordinate

The earlier expression for the geometrical optics force of a single ray (Eq. 3) was written in the ray frame of reference, the z–direction points (let us now rename this as z') in the direction of the incoming beam $(\hat{i})$ and y–direction (now y') is perpendicular to z in the plane of incidence. In our coordinate system, in the lab frame of reference, this can be written as,

$$\hat{\mathbf{z}}' = \hat{\mathbf{i}} = +\sin\theta\cos\phi\,\hat{\mathbf{x}} + \sin\theta\sin\phi\,\hat{\mathbf{y}} + \cos\theta\,\hat{\mathbf{z}} \tag{18}$$

and

$$\hat{\mathbf{y}}' = \frac{\hat{\mathbf{i}} \times (\hat{\mathbf{i}} \times \mathbf{r_0})}{|\hat{\mathbf{i}} \times (\hat{\mathbf{i}} \times \mathbf{r_0})|} \tag{19}$$

where we can use the identity $\hat{\mathbf{i}} \times (\hat{\mathbf{i}} \times \mathbf{r_0}) = \hat{\mathbf{i}}(\hat{\mathbf{i}} \cdot \mathbf{r_0}) - \mathbf{r_0}(\hat{\mathbf{i}} \cdot \hat{\mathbf{i}})$ to simplify the numerator as

$$\hat{\mathbf{y}}' = \frac{1}{\sqrt{r_0^2 - \xi^2}}((\xi\sin\theta\cos\phi - x_0)\,\hat{\mathbf{x}} + (\xi\sin\theta\sin\phi - y_0)\,\hat{\mathbf{y}} + (\xi\cos\theta - z_0)\,\hat{\mathbf{z}}) \tag{20}$$

where

$$\xi = z_0\cos\theta + \rho_0\sin\theta\cos(\phi - \phi_0) \tag{21}$$

Summing up all the rays, the resulting optical force in our coordinate system is,

$$\mathbf{F}_{\text{tot}} = \frac{n_i P_0}{c}\int_0^{\theta_{max}}\int_0^{2\pi} \sin\theta\cos\theta\,\mathrm{e}^{-\frac{\sin^2\theta}{f_0^2\sin^2\theta_{\max}}}\left\{[\mathrm{Re}\,\mathbf{f}, \mathrm{Im}\,\mathbf{f}] \cdot \left[\hat{z'}, \hat{y'}\right]\right\}\,\mathrm{d}\theta\mathrm{d}\phi \tag{22}$$

where,

$$\mathbf{f} = \left[1 + R\mathrm{e}^{2\mathrm{i}\sigma} - T^2 \mathrm{e}^{\mathrm{i}\alpha} \left(\frac{1}{1 - R\mathrm{e}^{\mathrm{i}\beta}} \right) \right] \tag{23}$$

is the complex force term from Eq. 3 for a single ray. The term in the curly brackets are a scalar product of the real part of the complex force term (Eq. 22) times $\hat{z}'$ of Eq. 18 plus the imaginary part times $\hat{y}'$ of Eq. 20.

Simplifications due to symmetries

Even though the previous equation is general we can still simplify it analytically for displacement along axis of symmetries.

z-axis

For beam displacements along the z-axis, we have $\rho_0 = 0$ and $r_0 = z_0$. When the sphere is trapped in a circular symmetric beam, it is expected that the resulting optical transverse forces are null. This results in the following expression for the incident angle from Eq. 17,

$$\cos\sigma = \sqrt{1 - \left(\frac{z_0 \sin\theta}{a} \right)^2} \tag{24}$$

for the condition of ray intercepting the sphere, the term inside the square root must be positive, i.e.,

$$\left(\frac{a}{z_0} \right)^2 > \sin^2\theta \tag{25}$$

or if $a < |z_0|$ then the θ–integration is limited to $\sin^{-1}(a/|z_0|)$, otherwise it is limited by the numerical aperture θ_{max}.

The mapping of the 2D vectors does not change for $\hat{\mathbf{z}}'$, but the $\hat{\mathbf{y}}'$ vector simplifies from Eqs.(19,20,21) to,

$$\hat{\mathbf{y}}' = \mathrm{sign}(z_0) \left(\cos\theta \cos\phi \, \hat{\mathbf{x}} + \cos\theta \sin\phi \, \hat{\mathbf{y}} - \sin\theta \, \hat{\mathbf{z}} \right) \tag{26}$$

Note that for the mapping vectors ($\hat{\mathbf{z}'}$,$\hat{\mathbf{y}'}$), the force $x-$ and $y-$component depends on $\cos\phi$ and $\sin\phi$, there upon integration from 0 to 2π, these terms are null as expected. This means that for the axial force component, for displacements along the z-axis we only need one integration.

x-axis

For beam displacements along the x-axis we have $\rho_0 = x_0$, $z_0 = 0$, and $\phi_0 = 0$ when x_0 is positive, and $\phi_0 = \pi$ when negative. This results in the following expression for the incident angle from Eq. 17,

$$\cos\sigma = \sqrt{1 - \left(\frac{x_0}{a}\right)^2 (1 - \cos^2\phi \sin^2\theta)} \tag{27}$$

for the condition of ray intercepting the sphere,

$$\left(\frac{a}{x_0}\right)^2 > 1 - \cos^2\phi \sin^2\theta \tag{28}$$

or, if $a < |x_0|$ then the θ–integration is limited to $\sin^{-1}\left(\frac{\sqrt{1-(a/x_0)^2}}{|\cos\phi|}\right)$, otherwise it is limited by the numerical aperture $\theta_{\max}$.

The $\hat{\mathbf{y}'}$ vector from Eqs.(19,20,21) simplifies to,

$$\hat{\mathbf{y}'} = \frac{\text{sign}(x_0)}{\sqrt{1 - \sin^2\theta\cos^2\phi}} \left\{(\sin^2\theta\cos^2\phi - 1)\,\hat{\mathbf{x}} + \sin^2\theta\cos\phi\sin\phi\,\hat{\mathbf{y}} + \sin\theta\cos\theta\cos\phi\,\hat{\mathbf{z}}\right\} \tag{29}$$

3. Neural Networks

In this work we have employed fully connected Neural Networks (NNs), which is one of the simplest deep learning models and has already been proved successful in similar regression problems.[8] In this section we will present the technical aspects that need to be considered.

Training

The training process consists of five main steps. The architecture definition and the data pre-processing, which are done only once, and the loading of the data, the training step, and the evaluation of the performance, that are carried out iteratively. The architecture definition consists of choosing the number of layers and the number of neurons per layer. A schematic of the structure of this type of NN can be found in Fig. 1 of the main manuscript. The architecture is adjusted according to the complexity of the different studied problems. In general, a higher number of trainable parameters will produce a model that will be able to learn more from the training data. However, we must be careful; we do not want to learn the artifacts coming from geometrical optics. The training data is obtained by calculating the optical forces using geometrical optics for a given set of parameters. These parameters can be spread over very different scales, from around unity in the case of parameters like the aspect ratio or the refractive index, to $\sim 10^{-6}$ for the positions, $\sim 10^{-12}$ for the forces and $\sim 10^{-18}$ for the torques. To achieve an efficient training of the NN we need to apply a pre-processing step where the variables must be rescaled around unity and the angles are expressed in terms of sines and cosines to avoid inconsistencies around 2π. Shuffling the data and dividing them into a validating and training set is the final step of the pre-processing.

The iterative part of the training starts by loading a subset of the training data and applying the training step where the NN weights are optimized to minimize the loss function. We used the mean squared error as the loss function and the Keras implementation of the Adam optimizer with the default parameters.[9] When the weights of the NN have been updated, the training data is deleted from the RAM memory and another subset of the training data is loaded before repeating the same process. Dividing the training set in smaller subsets (instead of loading all the data at once) allows to use big training sets independently of the RAM memory of the computer. Once the training dataset has been fully explored through all the subsets, the error between the NN calculation and the validating dataset (defined as the mean square difference) is computed. The iterative step is repeated until

this error stops decreasing. For the training data generated using for example 100 rays the artifacts are significant enough that a well trained NN could learn them. In order to prevent these artifacts from being included, the error between the NN calculations and the validating data generated with 1,600 rays is computed. The training stops when this value reaches its minimum. Fig. 2 in the main document shows how the NN starts to acquire the information of the artifacts present in the calculation with 100 rays. This is favoured by the fact that the architecture is more complex and by not using a validating data set generated with more rays to decide when to stop the training.

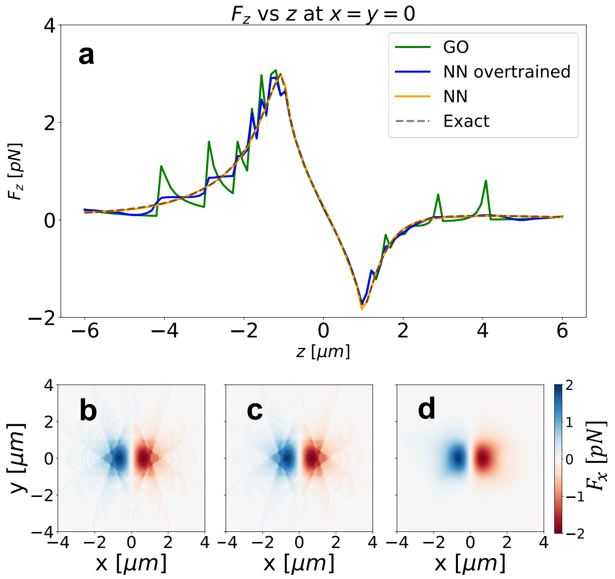

Fig. S2: Comparison between geometrical optics with 100 rays, the exact calculation, and two different NNs trained for 100 rays, one trained using a threshold and with a simple architecture (NN) and the other one trained without threshold and with a more complex architecture (NN overtrained). (a) Shows F_z in the axial direction with the different approaches. While the NN properly designed matches perfectly the exact calculation, the NN that is overtrained acquires the artifacts from geometrical optics. (b,c,d) show the force in the $x-y$ plane at $z = -1.2\mu$m calculated with geometrical optics, the overtrained NN, and the properly trained NN respectively. While the overtrained NN (c) keeps the overall structure of the artifacts, the properly trained NN (d) is able to remove them.

Architectures

Spheres (3 Degrees of freedom): The NN architecure for 100 and 400 rays consists of 5 hidden layers with 16 neurons each of them ($\approx 1 \cdot 10^3$ trainable parameters) while for 1600 rays consists of 3 hidden layers with 64 neurons in each of them ($\approx 8 \cdot 10^3$ trainable parameters)

Ellipsoids (9 Degrees of freedom): The NN architecture for 400 rays consists of 5 hidden layers with 384 neurons each of them ($\approx 6.0 \cdot 10^5$ trainable parameters) while for 1600 rays consists of 8 hidden layers with 384 neurons each ($\approx 1 \cdot 10^6$ trainable parameters)

Data generation

The data for training the NN is generated with the GO method described in Section 2.1 "Geometrical Optics". The optical forces calculation is computed with different numbers of rays, allowing to compare the machine learning and the traditional approaches in different situations. The data points are randomly selected from the parameter space of interest. Since the force profile changes faster close to the focus, the position parameters (x,y,z) are chosen according to a normal distribution centered in the beam focus. To get an uniform probability of orientations of the non-spherical particles in the case of the ellipsoids, the probability of generating an orientation angle θ is proportional to $\sin\theta$. The rest of the values of the parameters are equally probable.

Training region and range of validity of the NN

The training region and therefore the validity of the NN to compute optical forces is constrained to the given region of parameters. We believe that most of the experiments deal with situations contained in this region of parameters. Further training is required to compute optical forces outside of this region.

Table S1: **Parameters space for problems with 3 and 9 degrees of freedom**

	3 DOF	9 DOF
a	1μm	$[0.5\mu\text{m}, 3\mu\text{m}]$
c	1μm	$[0.5\mu\text{m}, 3\mu\text{m}]$
θ		$[0, \pi/2]$
ϕ		$[0, 2\pi]$
NA	1.3	$[0.25, 1.3]$
n	1.5	$[1, 4]$
$\mathbf{r}$	$x, y, z \in [-4\mu\text{m}, 4\mu\text{m}]$	$l = \max(a, c); x, y \in [-4l, 4l]; z \in [-6l, 6l]$

Hardware and software

The NNs are modelled and trained in Python using Keras (version 2.2.4-tf)[9] with TensorFlow backend (version 2.1.0). The training of the NN is done in a GPU type NVIDIA GeForce RTX 2060 with 16 GB of memory. The processor of the computer is an Intel Core i7-10700 and it has 16 GB of RAM.

4. Determination of the accuracy and the speed

To determine the accuracy of both the conventional geometrical optics and the NN methods we compare the force values calculated with each of the methods against our analytical model that served as gold standard. The error between the different approaches and the gold standard is determined as the average difference between the calculated force values.

To measure the computation speed, we used the same computer as in the training of the NN (see Supplement. 3, Neural Networks). Both geometrical optics and the NN are first compared in Matlab because the toolbox for the geometrical optics calculation[10] has been developed in this software. The calculation of the evaluation time using the GPU is done in Python for simplicity. We have defined the computation speed as the number of calculations that are carried out in the unit of time. This magnitude is determined by measuring the time that the different implementations need to calculate the forces at a given number of points.

5. Simulation of the Brownian dynamics of an ellipsoid in an optical field

The particle dynamics simulation consists on the integration of the Langevin equations considering the Brownian motion, the optical force and torque contribution, and the non spherical shape. Since we will be considering microscopic particles in water, we can safely consider the overdamped regime.[11] The diffusion tensor, which depends only on the shape of the particle, becomes slightly more complicated than in the case of a sphere but we can use the analytical solution for ellipsoids derived by Perrin.[12,13] While this tensor needs to be computed only once per simulation, the rest of the steps need to be computed iteratively.

In each time step we compute the contribution to the motion of the optical force and torque (lab frame of reference) and of the Brownian noise (particle frame of reference). Since both contributions are computed in different frames of reference, we need to continuously build the matrices that allow us to switch from one to another. While generating the Brownian motion contribution and computing the matrices can be fast, the optical contribution to the force and torque used to be the bottle neck of the process and is the one now being optimized by the NN. Once the contributions to the rotation and displacement are computed, we relocate and reorient the particle. To implement correctly the rotation of the axes of the particle reference frame we used the Rodrigues formula.[11,14] Repeating this process for each time step allows to construct the trajectories from where we can obtain statistical properties like the probability distribution or the Kramer's rate.

6. Trap stiffness dependence on the aspect ratio

We study how the trap stiffness changes with the aspect ratio of the ellipsoid. We keep all the parameters constant and change the long axis of the ellipsoid (c). The stiffness of the trap decreases when the aspect ratio increases, see Fig. S3.

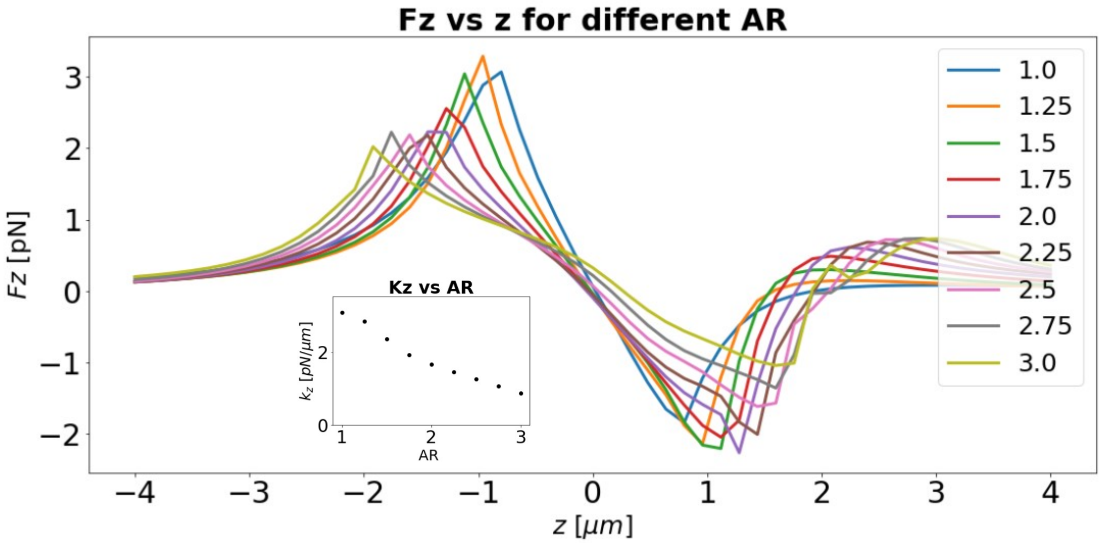

Fig. S3: Dependance of the trap stiffness on the aspect ratio. All the other parameters are kept constant. When the ellipsoids are longer, the trap stiffness is lower.

References

(1) Gieseler, J.; Gomez-Solano, J. R.; Magazzù, A.; Castillo, I. P.; García, L. P.; Gironella-Torrent, M.; Viader-Godoy, X.; Ritort, F.; Pesce, G.; Arzola, A. V., et al. Optical tweezers—from calibration to applications: a tutorial. *Advances in Optics and Photonics* **2021**, *13*, 74–241.

(2) Hänggi, P.; Talkner, P.; Borkovec, M. Reaction-rate theory: fifty years after Kramers. *Reviews of modern physics* **1990**, *62*, 251.

(3) Ashkin, A. Forces of a single-beam gradient laser trap on a dielectric sphere in the ray optics regime. *Biophysical Journal* **1992**, *61*, 569–582.

(4) Svoboda, K.; Block, S. M. Biological Applications of Optical Forces. *Annual Review of Biophysics and Biomolecular Structure* **1994**, *23*, 247–285, PMID: 7919782.

(5) Roosen, G.; Imbert, C. Optical levitation by means of two horizontal laser beams: A theoretical and experimental study. *Physics Letters A* **1976**, *59*, 6–8.

(6) Rocha, M. Optical tweezers for undergraduates: theoretical analysis and experiments. *American Journal of Physics* **2009**, *77*, 704–712.

(7) Fontes, A., et al. Uso de lasers para manipulação e medida de células vivas. **1999**,

(8) Lenton, I. C.; Volpe, G.; Stilgoe, A. B.; Nieminen, T. A.; Rubinsztein-Dunlop, H. Machine learning reveals complex behaviours in optically trapped particles. *Machine Learning: Science and Technology* **2020**, *1*, 045009.

(9) Chollet, F., et al. Keras: The python deep learning library. *Astrophysics Source Code Library* **2018**, ascl–1806.

(10) Callegari, A.; Mijalkov, M.; Gököz, A. B.; Volpe, G. Computational toolbox for optical tweezers in geometrical optics. *J. Opt. Soc. Am. B* **2015**, *32*, B11–B19.

(11) Callegari, A.; Volpe, G. Numerical simulations of active Brownian particles. *Flowing Matter* **2019**, 211.

(12) Perrin, F. Mouvement brownien d'un ellipsoide-I. Dispersion diélectrique pour des molécules ellipsoidales. *J. Phys. Radium* **1934**, *5*, 497–511.

(13) Han, Y.; Alsayed, A.; Nobili, M.; Yodh, A. G. Quasi-two-dimensional diffusion of single ellipsoids: Aspect ratio and confinement effects. *Physical Review E* **2009**, *80*, 011403.

(14) Dai, J. S. Euler–Rodrigues formula variations, quaternion conjugation and intrinsic connections. *Mechanism and Machine Theory* **2015**, *92*, 144–152.
